# Mechanism and Therapeutic Potential of Viral Mimicry in Cancer Immunotherapy

**DOI:** 10.3390/biom16050709

**Published:** 2026-05-12

**Authors:** Alisha Pearl Kirkland, Mahek Shah, Charles Spruck

**Affiliations:** Cancer Genome and Epigenetics Program, Sanford Burnham Prebys Medical Discovery Institute, La Jolla, CA 92037, USA

**Keywords:** cancer immunotherapy, repetitive elements, viral mimicry, innate immunity, adaptive immunity, epigenetic alteration, genomic instability, neoantigens, chimeric antigen receptor, natural killer cells, T cells, clinical trials

## Abstract

Cancer immunotherapy has transformed oncology by harnessing the immune system to recognize and eliminate malignant cells. However, many cancers exhibit limited or variable responses to this class of treatment due to insufficient antigen presentation and impaired interferon (IFN) signaling, creating an immunologically “cold” tumor microenvironment (TME) characterized by poor immune cell infiltration and treatment resistance. Viral mimicry has emerged as a therapeutic strategy to overcome these limitations by reactivating innate antiviral pathways within tumor cells. Viral mimicry occurs through the reactivation of endogenous retroviruses (ERVs) and other retrotransposons (e.g., LINE-1), which subsequently stimulate downstream nucleic acid sensing pathways. The resulting type I/III IFN responses restore antigen presentation and attract cytotoxic immune cells, sensitizing resistant tumors to immunotherapy. However, systemic stimulation of these pathways can trigger context-dependent inflammation and adaptive resistance, highlighting the need for temporal and spatial control. In this review, we examine the mechanistic foundation and clinical trajectory of viral mimicry, with an emphasis on its potential integration with established treatments and engineered immune cell platforms. By identifying the molecular and clinical gaps, viral mimicry can be harnessed to enhance tumor-specific immune activation and overcome treatment resistance in cancer immunotherapy.

## 1. Introduction

Cancer is the second leading cause of mortality worldwide, with global incidence projected to exceed 35.3 million cases by 2050 [1,2]. Over the past decade, immune checkpoint blockade (ICB) therapy has reshaped the treatment landscape by improving immune-mediated tumor surveillance. Following the U.S. Food and Drug Administration’s (FDA) approval of the first-in-class cytotoxic T lymphocyte antigen 4 (CTLA-4) checkpoint inhibitor for metastatic melanoma in 2011 [3], additional agents targeting programmed cell death protein 1 (PD-1), programmed cell death ligand 1 (PD-L1), and lymphocyte activation gene 3 (LAG-3) have produced durable clinical benefits across a range of malignancies [4]. Despite these advances, therapeutic responses remain highly variable and many patients experience immune-related adverse events (irAEs) resulting from systemic immune activation [5,6,7,8,9,10]. Moreover, emerging evidence suggests that sustained IFN signaling can promote immune dysfunction and resistance, further narrowing the therapeutic window of opportunity for ICB-based approaches. These limitations underscore the need for complementary strategies that enhance their efficacy while minimizing toxicity.

**Figure 1:**
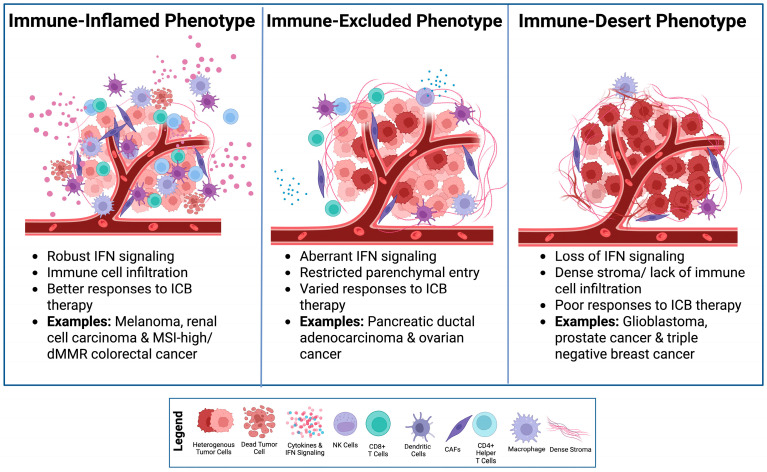
**Solid tumor immunophenotypes determine responsiveness to ICB therapy based on immune cell infiltration and IFN signaling.** Created in BioRender. Kirkland, A. (2026). https://BioRender.com/3ud0lsm (accessed on 27 March 2026).

One of the principal barriers to effective ICB therapy is an immunosuppressive TME. The TME is composed of a heterogeneous network of immune and stromal elements, including lymphocytes, cancer-associated fibroblasts (CAFs), endothelial cells, and extracellular matrix (ECM) components, that sustain tumor persistence and immune evasion [11]. Tumor-intrinsic features such as stromal density and vascular organization further restrict immune cell trafficking and nutrient accessibility, limiting effective immune engagement [12,13]. Based on the spatial distribution of tumor-infiltrating lymphocytes (TILs), tumors can be broadly categorized into immune-inflamed, immune-excluded, or immune-desert phenotypes [14,15,16,17] (Figure 1).

Inflamed tumors harbor cytotoxic CD8^+^ T cells and display IFN-γ signatures associated with improved responses to ICB therapy [18,19]. In contrast, immune-excluded tumors recruit immune cells but restrict their parenchymal entry through stromal barriers and aberrant chemokine gradients, whereas immune-desert tumors lack effective antigen priming and IFN signaling altogether [20,21,22,23,24,25]. Beyond these structural features, the TME is enriched with immunoregulatory cell populations, including regulatory T cells (Tregs) and myeloid-derived suppressor cells (MDSCs), that suppress cytotoxic lymphocyte activity [25,26]. At the molecular level, epigenetic repression of immune-related genes further constrains anti-tumor immunity. In triple negative breast cancer, a hypoxic TME drives epigenetic suppression of cytokines such as TNF-α and IFN-γ, which contributes to T cell and natural killer (NK) cell dysfunction and promotes resistance to immunotherapy [22,23].

Together, these structural and epigenetic barriers render tumors poorly visible to immune recognition and promote immunologically “cold” states that are refractory to ICB therapy. Overcoming immune exclusion therefore requires strategies that can restore IFN responsiveness while avoiding chronic or dysregulated activation. One emerging approach to achieve this is the induction of viral mimicry, in which normally silenced retroelements embedded in the non-coding genome are transcriptionally reactivated to generate cytosolic double-stranded RNA (dsRNA) and double-stranded DNA (dsDNA) that resemble viral genetic material. These nucleic acids engage pattern-recognition receptors (PRRs), which are responsible for identifying infections and initiating type I and III IFN responses, leading to enhanced antigen presentation and immune signaling. However, the magnitude, duration, and spatial restriction of this response remain critical determinants of therapeutic efficacy and toxicity. Emerging evidence indicates that pairing viral mimicry with ICB therapy can improve treatment efficacy in tumors refractory to immunotherapy [24,27,28]. In preclinical models, viral mimicry-inducing epigenetic agents increase IFN-driven immune infiltration and enhance cytotoxic T cell and NK cell activity, supporting their role as sensitizers within immunologically “cold” TMEs [29,30].

This review examines how viral mimicry links epigenetic regulation to innate immune activation to restore tumor visibility within an immunosuppressive TME. It further outlines the core mechanisms that define viral mimicry and considers how pharmacological inducers can remodel immune responses in clinical settings. An emphasis is placed on the therapeutic constraints that shape this approach and emerging strategies to integrate viral mimicry with ICB therapy and engineered cell therapies. We further evaluate how viral mimicry-driven upregulation of antigen presentation enhances tumor recognition by T cells and NK cells, thereby improving the efficacy of adoptive cell therapies, while addressing resistance mechanisms that limit durable responses.

## 2. Repetitive Element Activation and Innate Immune Signaling

The human genome harbors multiple classes of repetitive elements (REs) that require tightly regulated epigenetic control to maintain chromatin integrity and prevent aberrant immune activation. When this repression is lifted, these elements can generate nucleic acid intermediates that resemble viral genomes and engage innate immune sensing pathways. This process underlies viral mimicry; however, its biological consequences are highly context-dependent. The immunological consequences of RE derepression are therefore dictated by the classes of REs involved and the magnitude of downstream signaling.

### 2.1. Classes of REs

REs are a defining feature of eukaryotic genomes and play a central role in shaping genomic architecture and regulatory complexity. In humans, REs constitute nearly half of all genomic DNA and were once described as “junk DNA”. However, emerging studies have shown that these sequences play active roles in chromatin organization and gene regulation [31,32]. Based on structure and genomic distribution, REs are broadly categorized into tandem repeats and interspersed repeats.

Tandem repeats consist of sequence motifs arranged at defined chromosomal loci and are classified as microsatellites, minisatellites, and satellite DNA [33]. Microsatellites comprise short repeat units of 1 to 6 base pairs and primarily serve structural and genomic functions. Minisatellites and satellite DNA contribute to telomeric and centromeric architecture to support chromosomal stability [34,35,36,37]. Although largely non-coding, tandem repeats contribute to nuclear organization and have not been shown to activate viral mimicry pathways. Instead, their dysregulation is associated with genomic instability and repeat expansion disorders such as Huntington’s disease and myotonic dystrophy [38,39,40].

In contrast, interspersed repeats are distributed throughout the genome and primarily originate from transposable elements (TEs) [41]. These include retrotransposons, which propagate through RNA intermediates, and DNA transposons, which mobilize directly at the DNA level [42,43]. While these elements have been a major driver of genomic diversity over evolution, their mobilization introduces mutagenic risk and requires strict epigenetic repression [44,45].

Retrotransposons represent the dominant class of interspersed REs and include LINEs, SINEs, and ERVs. LINEs, particularly LINE-1 (L1), are the only autonomous retrotransposons that remain active in humans and encode endonuclease and reverse transcriptase activities required for their mobilization and genomic reintegration [46]. Although only a fraction of the roughly 500,000 L1 copies are fully functional, active retrotransposition events have been documented in both germline and somatic tissues, including during neuronal development and cancer [47,48,49]. Aberrant L1 activation can disrupt coding regions and regulatory landscapes, contributing to genomic instability and oncogenesis [50].

SINEs are non-autonomous retrotransposons that rely on L1 machinery for mobilization, and typically range from 100 to 400 bases in length [51]. Alu elements are the most abundant SINEs, comprising approximately 11% of the human genome. Despite lacking self-replication capacity, their widespread insertion shapes transcriptional regulation and contributes to gene expression variability [52,53].

ERVs represent remnants of ancient retroviral infections that integrated into germline DNA millions of years ago and became vertically inherited [54]. Although most human ERVs are defective due to accumulated mutations and truncations, they retain substantial regulatory potential. The long terminal repeats (LTRs) of ERVs can function as alternative promoters or enhancers, as observed in processes such as placental development [55], where tightly regulated ERV activation supports tissue-specific gene expression programs. In pathological contexts, including cancer and aging, loss of ERV silencing can result in the accumulation of viral-like transcripts that activate innate immune pathways. This reflects a shift from regulated transcriptional activity to aberrant immunostimulatory signaling, and forms the central mechanism of viral mimicry discussed later in this review [56].

Together, tandem and interspersed repeats establish an architectural and regulatory framework that supports genome stability while permitting adaptive flexibility. While tandem repeats primarily support structural genome organization, interspersed repeats contribute to regulatory diversity through transcriptional activity. This balance is maintained by epigenetic mechanisms that silence REs in somatic cells through DNA methylation and histone modifications [57].

### 2.2. Derepression and Transcriptional Reactivation of REs

REs remain transcriptionally silenced in healthy cells through a network of epigenetic pathways that stabilize chromatin and limit aberrant transcription. DNA methylation, maintained by DNA methyltransferases, restricts access to retrotransposon promoters and preserves compact chromatin through the addition of a methyl group to the fifth carbon of cytosine residues (5-methylcytosine) at CpG dinucleotides [58,59]. Histone methyltransferases such as SUV39H1 and SETDB1 reinforce this repression through histone H3 Lys9 trimethylation (H3K9me3) across endogenous retroviral and L1 loci [60,61]. Small RNA-mediated pathways, including piRNA and siRNA systems, provide additional control by directing newly synthesized transcripts toward nuclear retention or degradation to prevent the accumulation of immunostimulatory RNA species [62]. Through these coordinated mechanisms, the generation of viral-like nucleic acids is normally suppressed to maintain immune tolerance to endogenous genomic elements.

Disruption of epigenetic silencing mechanisms can reactivate RE transcription and produce viral intermediates. Bidirectional transcription at derepressed loci generates complementary RNA species that anneal into double-stranded (ds) structures that can be detected by cytosolic dsRNA sensors [27,63]. Additional sources of dsRNA include inverted repeat transcripts, aberrant RNA processing, and mitochondrial dsRNA release [64,65,66,67]. Under normal conditions, these transcripts are retained in the nucleus or eliminated by quality-control pathways. However, when transcriptional output increases beyond this capacity, dsRNA can accumulate in the cytoplasm [68].

Elevated L1 expression further contributes to this process by increasing the activity of its encoded reverse transcriptase and generating cDNA intermediates that exceed the degradative capacity of TREX1 and related cytosolic exonucleases responsible for processing DNA generated during genotoxic stress [69,70]. The combined accumulation of cytosolic dsRNA and dsDNA establishes a molecular profile that resembles viral infection and initiates innate immune sensing.

### 2.3. Activation of Cytosolic Nucleic Acid Sensing Pathways

**Figure 2:**
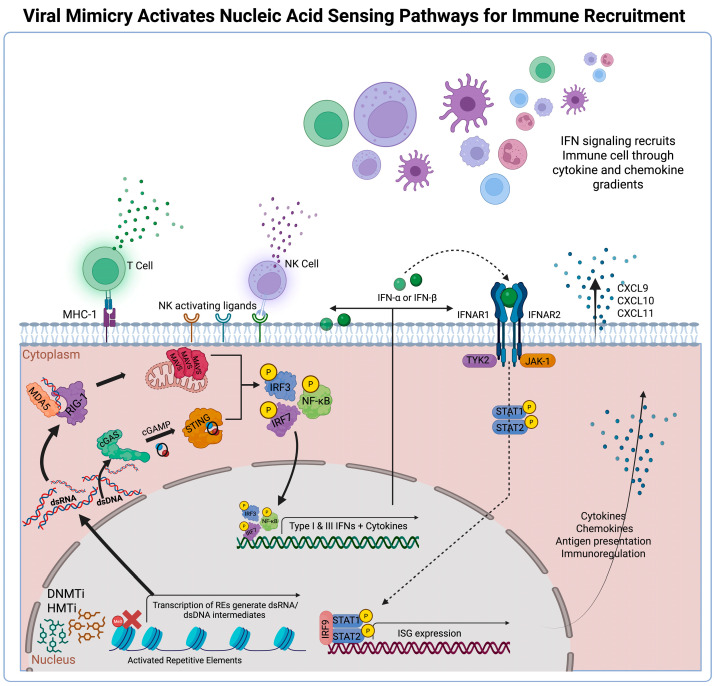
**Viral mimicry activates the cytosolic nucleic acid sensing cascade, recruiting immune cells.** Cytosolic nucleic acids activate RIG-I, MDA5, and cGAS–STING pathways to trigger type I/III IFN responses, leading to enhanced chemokine production, antigen presentation, and recruitment of innate and adaptive immune cells into the TME. Created in BioRender. Kirkland, A. (2026). https://BioRender.com/ud78d3b (accessed on 27 March 2026).

Derepression of REs generates nucleic acids that enter the cytoplasm and engage PRRs to coordinate antiviral signaling (Figure 2). This mechanism positions nucleic acid sensing as a determinant of tumor immunogenicity, especially in tumors with limited immune infiltration, where defects in innate sensing contribute to an immunologically cold TME that resists immune clearance [71].

Cytosolic dsRNA is detected by multiple innate immune sensors including the RIG-I–like receptors, protein kinase R, oligoadenylate synthetases, and endosomal Toll-like receptor 3 [72,73,74,75]. These innate immune pathways initiate antiviral responses through IFN production and RNA degradation. Among these sensors, retinoic acid-inducible gene-I (RIG-I) and melanoma differentiation-associated protein 5 (MDA5) serve as principal mediators of viral mimicry. RIG-I recognizes short duplex RNAs bearing di- or triphosphate termini, whereas MDA5 assembles along extended dsRNA substrates to form filaments that promote receptor activation [76,77]. Signaling by either receptor proceeds through oligomerization of their caspase activation and recruitment domains (CARDs), enabling recruitment of mitochondrial antiviral-signaling protein (MAVS) to the mitochondrial outer membrane [78]. MAVS polymerization establishes a signaling platform that positions TANK-binding kinase 1 (TBK1) and IκB kinase (IKK) in proximity to their substrates. This organization supports TBK1-mediated phosphorylation of interferon regulatory factor 3 (IRF3) and interferon regulatory factor 7 (IRF7) to induce IFN responses, while IKK activation promotes nuclear factor-κB (NF-κB)-dependent inflammation [79,80].

L1 reverse transcription generates cytosolic dsDNA that activates the cyclic GMP-AMP synthase (cGAS)–stimulator of interferon genes (STING) pathway. cGAS binds dsDNA in a length-dependent manner and promotes synthesis of cyclic GMP-AMP (cGAMP) [81,82]. cGAMP then activates STING at the endoplasmic reticulum, driving STING oligomerization and trafficking toward the Golgi, where it recruits TBK1 to promote IRF3 phosphorylation and modulate NF-κB activity as part of the antiviral response [83,84,85].

Cytosolic dsRNA- and dsDNA-sensing pathways converge on shared downstream modules that amplify innate immune activation following RE derepression. These pathways induce IFN-stimulated transcriptional programs that increase stress-induced ligand expression on tumor cells and support infiltration of cytotoxic lymphocytes [86,87]. Through these mechanisms, cytosolic nucleic acid sensing increases tumor visibility within immunologically cold TMEs and may help overcome resistance to ICB therapy.

### 2.4. Innate Immune Recruitment and Tumor Visibility

Cytosolic nucleic acid sensing initiates a transcriptional response driven by type I/III IFNs and NF-κB-dependent cytokines that together establish an antiviral state within tumor cells [88] (Figure 2). Type I IFNs signal through the Janus kinase–signal transducer and activator of transcription (JAK-STAT) pathway by activating JAK1 and tyrosine kinase 2 (TYK2) to phosphorylate the transcription factors STAT1 and STAT2. These transcription factors associate with IRF9 to form the interferon-stimulated gene factor 3 (ISGF3) complex. This complex enters the nucleus and binds IFN-stimulated response elements, driving the expression of interferon-stimulated genes (ISGs) [89,90]. Engagement of these elements regulates antigen processing and presentation while enhancing immune cell recognition [91,92,93].

IFN signaling also increases the density of peptide–major histocompatibility complex (MHC) displayed at the tumor cell surface, which presents tumor-derived peptides to CD8^+^ T cells within the viral mimicry-induced TME [94]. IFN responses also induce stress-associated ligands, which engage the natural killer group 2D receptor (NKG2D) on cytotoxic NK cells and promote their activation [95,96]. Through these coordinated efforts, these pathways provide MHC class I-dependent and -independent mechanisms that broaden immune recognition of tumor cells undergoing viral mimicry, as discussed in Section 4.1 and Section 4.2 below. Understanding how to exploit these interactions will inform the development of viral mimicry–based therapies for treatment-resistant tumors.

The therapeutic potential of viral mimicry on immune cell infiltration depends on the duration and magnitude of IFN signaling. Transient activation establishes a localized antiviral state that elevates ligand expression and drives chemokine expression, which recruits and sustains cytotoxic lymphocytes within the tumor [97,98]. However, prolonged IFN exposure disrupts cytokine balance and promotes functional exhaustion of both T cells and NK cells [99,100,101]. Sustained signaling selects for tumor cell variants with impaired JAK-STAT responsiveness or defects in IFN signaling, reducing antigen presentation and weakening immune surveillance [102]. The therapeutic outcome of viral mimicry therefore hinges on whether RE activation produces a temporally restricted antiviral response that supports immune clearance or a persistent inflammatory state that facilitates immune escape.

## 3. Therapeutic Induction of Viral Mimicry

### 3.1. Pharmacologic and Synthetic Inducers

Epigenetic therapies that induce RE derepression have emerged as tools for increasing tumor antigenicity and sensitizing tumors to immunotherapy. DNA methyltransferase inhibitors (DNMTi) provided early clinical evidence linking epigenetic modulation to immune engagement. Azacitidine and decitabine were approved in the early 2000s for the treatment of myelodysplastic syndrome (MDS), establishing hypomethylating agents as a therapeutic class in hematologic malignancies [103]. Second-generation analogues, including the decitabine prodrug guadecitabine [104] and non-nucleoside DNMT1-selective inhibitors such as GSK3685032 [105], were subsequently developed to achieve more durable hypomethylation with improved clinical tolerability in acute myeloid leukemia models [106,107]. Guadecitabine has since entered multiple clinical studies across hematologic and solid tumors, including in combination with PD-L1 blockade in metastatic urothelial carcinoma and dual CTLA-4/PD-1 blockade in melanoma [106,107,108].

At the molecular level, DNMTi derepress ERVs and generate dsRNA that activates type I IFN programs and sensitizes tumors to ICB therapy [27]. This principle extends across multiple tumor types. In renal cell carcinoma, DNMTi treatment promoted TE expression and innate immune signaling. Additionally, studies in ovarian and cervical cancer models showed that this response restored TH1-associated chemokine expression (CXCL9, CXCL10), strengthened IFN signatures, and increased cytotoxic T cell recruitment [109,110,111]. In murine melanoma, guadecitabine induced widespread DNA demethylation and improved the efficacy of combined CTLA-4 and PD-1 blockade [108]. This response was linked to increased MHC class I expression on tumor cells, expansion of cytotoxic CD8^+^ T cell and NK cell activity, and reduced suppressive Treg and MDSC populations within the TME.

Recognition that DNA hypomethylation can engage antiviral pathways prompted the investigation of histone methyltransferases as complementary regulators of viral mimicry. H3K9me3 represents a dominant repressive mark at ERV and LINE-1 loci, with enzymes such as SUV39H1 and SETDB1 acting as key regulators of these domains [60]. In breast and colon cancer models, genetic or pharmacologic inhibition of SUV39H1 reduced H3K9me3 at REs, permitting ERV transcription and accumulation of cytosolic nucleic acids that amplify IFN pathway activation, demonstrating that histone methylation also constrains viral mimicry [112,113,114]. Beyond tumor cells, SUV39H1 activity in tumor-infiltrating CD8^+^ T cells limits effector differentiation and contributes to resistance to PD-1 blockade. Loss of SUV39H1 restored effector gene expression and improved checkpoint responsiveness in melanoma models, linking epigenetic repression in T cells to immunotherapy efficacy [112,113,114]. SETDB1 has similarly emerged as a regulator of tumor immunogenicity. CRISPR-based screens in melanoma identified SETDB1 and components of the human silencing hub (HUSH) complex as central silencers of viral mimicry. SETDB1 loss induced type I IFN signaling, enhanced antigen presentation, and supported CD8^+^ T-cell-dependent tumor clearance, showing that release of this repression increases tumor visibility and enables effective immune clearance [115].

Additional chromatin regulators further refine this axis. Histone deacetylase (HDAC) inhibitors increase chromatin accessibility and can cooperate with DNMTis to strengthen ERV transcription and IFN induction, although their effects are broader and less specific to REs [116]. Combined DNMT and HDAC inhibition generates polyadenylated ERV-derived transcripts that are processed into neoantigenic peptides and presented on MHC class I, directly linking viral mimicry to treatment-induced neoantigen formation [116]. EZH2 inhibitors relieve H3K27me3-mediated repression at loci that remain silenced following DNMTi treatment. In colon cancer models, select EZH2 inhibitors potentiated DNMTi-induced viral mimicry by counteracting compensatory H3K27me3-mediated repression and engaging NFAT–AP-1 signaling [117]. This finding supports combination epigenetic targeting as a strategy to deepen viral mimicry responses when DNMT inhibition alone is limited by compensatory chromatin repression.

LSD1 plays multiple roles in retroviral silencing through H3K9me2 demethylation and stabilization of the RNA-induced silencing complex (RISC), which degrades aberrant cytosolic dsRNA. Therefore, LSD1 inhibition promotes ERV transcription and dsRNA accumulation [118]. Other regulators, including demethylase PHF8 and H3K9 methyltransferase G9a (also called EHMT2), also constrain retrotransposon expression and IFN responses. Loss or inhibition of these enzymes activated ERV-driven antiviral programs, promoted durable antitumor immune memory, and increased sensitivity to ICB therapy in preclinical models [119,120,121]. These findings collectively indicate that both DNA and histone methylation pathways can be manipulated to control the persistence and magnitude of viral mimicry in tumors for therapeutic potential.

Synthetic agonists of PRRs provide an orthogonal strategy to induce viral mimicry by supplying defined viral-like nucleic acid signals without requiring endogenous RE derepression (Table 1). Poly(I:C), a synthetic double-stranded RNA analogue, activates TLR3 and MDA5, drives type I IFN production, and promotes cytotoxic T cell priming [122]. In mouse models, poly(I:C) improved T cell tumor infiltration and strengthened responses to vaccines and checkpoint blockade, while poly-ICLC has been evaluated in glioblastoma (GBM) as an immune adjuvant paired with vaccination or ICB therapy [123,124]. These studies position synthetic dsRNA agonists as immune primers that can restore inflammatory tone in tumors with weak endogenous sensing.

RIG-I agonists, such as 5′-triphosphate RNAs and structured ligands such as SLR14, mimic viral RNA termini to activate RIG-I and promote dendritic cell activation and cross-priming [124,125,126,127]. In syngeneic tumor models, intratumoral SLR14 induced robust anti-tumor responses, while nanoparticle delivery of 5′-triphosphate RNA improved RIG-I activation and sensitized tumors to PD-1 blockade [124,125,126]. The benefit of RIG-I agonism therefore depends not only on RNA sensing but also on delivery strategies that concentrate activation within the TME.

Synthetic STING agonists, such as ADU-S100, mimic cyclic dinucleotides to activate cGAS–STING signaling downstream of cytosolic DNA sensing, promoting CD8^+^ T cell priming and tumor regression in mouse models [128]. However, STING agonist efficacy depends on tumor-intrinsic STING expression and epigenetic state, with monotherapy often failing to sustain durable tumor control [129]. These observations show that synthetic viral mimicry platforms offer controllable viral-like danger signals, but their therapeutic value depends on matching the agonist, delivery route, and tumor sensing state.

Beyond soluble PRR agonists, virus-inspired engineering offers additional control over how viral-like nucleic acid sensing is initiated. Synthetic RNA or DNA mimetics can be designed to engage defined PRRs, while self-amplifying and self-replicating RNA systems encode replicase machinery that amplifies RNA within target cells and increases antigen expression at lower input doses [130,131]. Engineered virus-like particles add another layer of control by packaging mRNA, protein, or ribonucleoprotein cargo into noninfectious structures that retain selected features of viral delivery without productive viral replication [132,133]. These platforms shift therapeutic design from broad pharmacologic derepression toward engineered delivery of viral-like signals, where dose, cargo, tissue distribution, and persistence can be tuned. Their translational value will depend on whether nucleic acid sensing can be concentrated within tumor compartments, since the same features that strengthen IFN induction may increase systemic inflammation when delivery is poorly controlled.

### 3.2. Temporal Dynamics and Immune Consequences

The kinetics of nucleic acid sensing differ fundamentally between epigenetic inducers and synthetic PRR agonists, shaping both immune activation and therapeutic risk.

Synthetic PRR agonists are administered at defined dosing intervals, either intratumorally or systemically, triggering rapid bursts of type I/III IFN [71]. Type I IFN produces a potent but rapidly restrained inflammatory response, while type III IFN emerges more slowly and persists longer, resulting in distinct temporal profiles of immune activation [134]. Together, these responses generate an acute but transient antiviral state. Because signaling resolves quickly, PRR agonists are often delivered locally to concentrate inflammation within the tumor and limit systemic toxicity. This temporal control is therapeutically attractive, but it also makes efficacy dependent on dose, route, and the ability to reach immune-relevant cells within the TME.

Epigenetic agents operate on slower timescales and generate more durable responses. DNMTis and histone-modifying drugs alter chromatin structure and derepress REs, causing sustained accumulation of endogenous dsRNA and prolonged activation of ISGs [27]. In cervical cancer models, decitabine induced dsRNA buildup and maintained IFN-associated transcriptional programs for more than a week after drug withdrawal [135]. Prolonged signaling improved antigen presentation and increased stress-induced ligand expression at the tumor surface. However, sustained IFN exposure also creates selective pressures that can shape tumor evolution. Thus, the same durability that makes epigenetic viral mimicry attractive can also narrow its therapeutic window.

Tumor adaptation is strongly shaped by regulators of nucleic acid metabolism that set the threshold for antiviral signaling. ADAR1 edits endogenous dsRNA and limits aberrant RNA sensing, while ADAR1 loss heightens tumor sensitivity to IFN signaling and ICB therapy [136]. TREX1 similarly restricts cGAS–STING activation by degrading cytosolic DNA generated during genotoxic stress, and elevated TREX1 expression has been linked to reduced IFN signaling and resistance to radiotherapy-induced immunogenicity [137]. These pathways can blunt viral mimicry or select for tumor states with impaired nucleic acid sensing, reinforcing the need for temporal control and tumor-restricted activation. Emerging approaches pair viral mimicry inducers with engineered cell therapies to expand tumor visibility and improve immune-mediated clearance (Table 1).

## 4. Impact of Viral Mimicry on the Immune System and Cell Therapies

Viral mimicry has therapeutic value because it can convert tumor-intrinsic nucleic acid sensing into immune-visible changes across the TME. These changes can support antigen presentation, immune trafficking, tumor–immune contact, and stress ligand recognition, making them particularly relevant in immune-excluded and immune-desert tumors. However, the same IFN-driven programs that promote tumor visibility can drive exhaustion and resistance when signaling becomes chronic. This section examines how viral mimicry reshapes innate and adaptive immunity, and where this remodeling creates therapeutic opportunities or constraints.

### 4.1. Immune Remodeling by Viral Mimicry

Viral mimicry alters tumor–innate interactions through coordinated changes in antigen presentation, immune trafficking and tumor–immune adhesion [91,92,93]. IFN-stimulated transcription increases MHC class I expression and enhances peptide loading through TAP1, TAP2, and β2-microglobulin, raising MHC-I density at the tumor surface. This strengthens CD8^+^ T cell receptor engagement.

In parallel, IFN signaling induces chemokines such as CXCL9, CXCL10, and CXCL11, which support the recruitment of CD8^+^ T cells and NK cells into tumors [94]. These chemokine gradients are often weak or absent in immune-desert and immune-excluded tumors, where effective immune surveillance is impeded by low signaling. Increased chemokine expression therefore restores effector cell entry and repeated tumor–immune contact [138].

**Figure 3:**
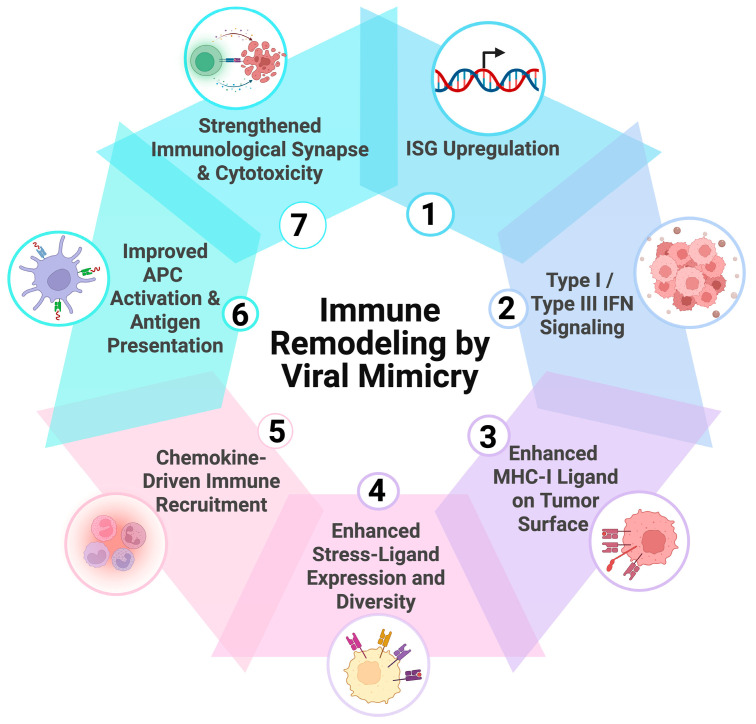
**Immune remodeling by viral mimicry**. Viral mimicry links IFN-stimulated transcription to tumor visibility by coordinating antigen presentation, stress-ligand expression, immune recruitment, antigen-presenting cell (APC) activation, and tumor–immune contact. The numbered cycle summarizes major innate and adaptive remodeling events within the tumor microenvironment. Created in BioRender. Kirkland, A. (2026). https://BioRender.com/3ud0lsm (accessed on 27 March 2026).

IFN-dependent transcription also upregulates adhesion molecules, including intercellular adhesion molecule 1 (ICAM1), which facilitates lymphocyte transmigration and stabilizes immunological synapse formation once effector cells reach the tumor parenchyma [139,140]. By increasing antigen display and promoting immune cell recruitment, viral mimicry supports cytotoxic engagement at the tumor surface (Figure 3).

Immune remodeling, however, is not uniformly beneficial. Sustained IFN exposure activates compensatory pathways that limit the therapeutic impact of viral mimicry. Prolonged type I IFN exposure promotes negative feedback within the JAK–STAT axis and may select for tumors with reduced IFN responsiveness [141]. Tumors previously exposed to immunotherapy frequently exhibit impaired JAK–STAT signaling, which diminishes sensitivity and can increase inhibitory mediators such as PD-L1 and IDO1, reducing T cell recruitment and promoting resistance to ICB therapy [142,143]. Clinical findings align with this model. Studies evaluating hypomethylating agents in combination with PD-L1 blockade report stronger responses in treatment-naïve patients, whereas heavily pretreated tumors demonstrate attenuated IFN signaling and reduced lymphocyte infiltration [144].

The central therapeutic challenge is to induce viral mimicry in a manner that improves immune infiltration without sustaining inflammatory signaling that promotes immune escape. Defining these constraints provides a framework for integrating viral mimicry with immunotherapies that require coordinated innate and adaptive engagement, including engineered cell-based approaches.

### 4.2. Innate-to-Adaptive Immune Priming by Viral Mimicry

Viral mimicry can function therapeutically by restoring inflammatory signals that are weak or absent in immune-excluded and immune-desert tumors. Innate effector cells, including NK cells, dendritic cells, macrophages, and innate lymphoid populations, respond rapidly to inflammatory cues and coordinate early immune activation [141,142]. In tumors with poor chemokine expression or stromal barriers that restrict immune-cell entry, this early inflammatory phase is often insufficient to support immune surveillance [19,143]. Viral mimicry addresses this deficit by generating endogenous nucleic acid ligands that activate RIG-I, MDA5, and cGAS–STING pathways within tumor cells [145,146,147,148]. Tumor-intrinsic sensing drives local type I/III IFN and inflammatory cytokine production, aligning viral mimicry with early steps of the Cancer Immunity Cycle, in which innate activation precedes effective immune engagement [149,150].

NK cells are a major early target of this IFN-conditioned environment. NK cell activation depends on the balance between inhibitory signaling through MHC class I molecules and activating signals provided by stress-induced ligands [151]. Viral mimicry shifts this balance by increasing surface ligands such as MICA, MICB, ULBP2, ULBP5, ULBP6, CD112, and CD155, which engage activating receptors such as NKG2D and DNAM-1 on NK cells [152,153]. IFN signaling also increases ICAM-1, Fas, and TRAIL-R expression, strengthening tumor–NK cell contact and cytotoxic activity [154,155]. These effects are especially relevant in immune-poor tumors, where NK cells may represent early infiltrating lymphocytes capable of IFN-γ production and granzyme-mediated killing [156,157].

Activation of IFN pathways downstream of viral mimicry also reshapes the APC and myeloid compartments. Type I/III IFNs promote dendritic cell maturation and MHC class II expression to enhance cross-presentation capacity [158]. IFN-stimulated chemokines recruit dendritic cells into tumors to support antigen acquisition and migration to lymphoid tissues, resulting in activation of adaptive immune responses [159,160]. Macrophages respond in parallel, as IFN signaling favors pro-inflammatory polarization and limits the expansion of myeloid-derived suppressor populations [161,162]. In GBM models, STING activation has also been shown to remodel the tumor vasculature to increase permeability and facilitate immune cell trafficking into tumor tissue [163]. Together, these innate effects create the inflammatory context needed for adaptive immunity.

These innate effects are constrained by feedback mechanisms. Sustained IFN exposure induces inhibitory ligands such as HLA-E on tumor cells that engage inhibitory receptors, such as NKG2A, on NK cells and promote functional impairment [164]. Similar counter-regulatory pathways can dampen nucleic acid sensing and reduce responsiveness to continued inflammatory stimulation. The magnitude and duration of activation therefore determine whether viral mimicry supports productive engagement or promotes regulatory adaptation. This balance sets the conditions for adaptive immunity, where effective T cell priming depends on whether innate activation produces durable tumor visibility rather than transient inflammation.

Adaptive immunity depends on whether innate activation is translated into durable antigen presentation and T cell priming [165]. Following viral mimicry induction, dendritic cells cross-present tumor-associated antigens and support expansion of tumor-reactive T cell populations [163,164]. CD4^+^ helper T cells reinforce APC function while CD8^+^ cytotoxic T cells recognize peptide–MHC class I complexes on tumor cells and mediate targeted cytotoxicity [166,167,168]. These responses require intact antigen processing, co-stimulatory signaling, and effector cell access to the TME [169]. Tumors frequently disrupt this sequence through MHC class I downregulation and immunosuppressive niches that limit T cell recruitment and function [170,171].

Viral mimicry partially restores these disrupted processes by expanding antigen availability and tumor surface presentation. ERV-derived peptides are processed and displayed on MHC class I molecules in ovarian and hematologic malignancies, where they can be recognized by tumor-reactive T cells and serve as targets for T cell–based immunotherapy [172,173]. IFN signaling further strengthens this process by increasing antigen processing and MHC class I expression, raising the density of peptide–MHC complexes accessible to CD8^+^ T cells [174]. Together, these effects increase the probability that tumor-reactive T cells can recognize and engage malignant cells.

Adaptive immune engagement also depends on coordination with innate effector populations. Early NK cell activation promotes dendritic cell maturation and contributes to tumor debulking, which can limit anergic T cell states [175]. Chemokine-driven recruitment of CXCR3^+^ effector T cells into previously inaccessible tumor regions allows ICB therapy, including PD-1 and CTLA-4 inhibition, to function in the presence of tumor-infiltrating lymphocytes [138,176,177]. In this context, viral mimicry may convert immune-excluded and immune-desert tumors into states more permissive to ICB and T cell–based therapies.

These viral mimicry-associated benefits can be limited by prolonged inflammatory signaling. Chronic type I IFN exposure induces co-inhibitory receptors, such as PD-1, TIM-3, and LAG-3, on T cells and NK cells, reducing cytotoxic lymphocyte function [99,100]. A sustained inflammatory state also favors recruitment of Tregs and MDSCs, reinforcing immunosuppression [178,179]. Temporal control of viral mimicry induction is therefore critical to limit adaptive resistance. Strategies that restrict IFN duration or combine viral mimicry with ICB therapy may preserve antigen expansion and effector infiltration while reducing exhaustion and immune suppression.

## 5. Clinical Translation and Emerging Strategies

### 5.1. Preclinical Mechanisms of Hypomethylating Agents

Although initially developed as a method of tumor suppressor silencing reversal, hypomethylating agents (HMAs) have now been established as potent inducers of viral mimicry through extensive preclinical work. Mechanistic evidence for viral mimicry induction by HMAs is supported across multiple tumor types. In colorectal cancer precursor cells, low-dose decitabine generated cytosolic dsRNA that activated the MDA5–MAVS pathway, consistent with canonical RNA-sensing antiviral signaling [27]. In renal cell carcinoma, HMA-mediated hypomethylation increased TE expression and activated IFN pathways without triggering somatic retrotransposition [109]. In murine mammary tumor models, decitabine activated the expression of ERV loci, including mouse mammary tumor virus (MMTV), leading to IFN-β induction and suppression of tumor proliferation [180]. Although MMTV is typically regarded as a provirus that contributes to mammary tumor formation, its re-expression in this study led to a robust IFN-β response, which outweighed tumor-promoting effects. Furthermore, studies in myeloid malignancies similarly demonstrated increased L1 and related retroelement transcripts without evidence of new genomic insertions, indicating that viral mimicry signaling may be decoupled from insertional mutagenesis risk [181].

HMAs have also been shown to modulate the surface repertoire of different cancer types. In GBM cells, decitabine increased neoantigen and cancer testis antigen (CTA) expression, enhancing MHC-I presentation and improving T cell–mediated toxicity [182]. Similarly, various ovarian cancer cell lines treated with decitabine were found to upregulate expression of 11 CTA genes as well as to show an overall increase in MHC-I presentation [183]. These findings support decitabine and other HMAs as an avenue of novel target identification and expression.

HMAs also modulate adaptive immune responses. Low-dose decitabine increased IFN-γ^+^ T cells and promoted Th1 polarization, correlating with improved antitumor responses [184,185]. Decitabine enhanced NF-κB signaling in CD4^+^ T cells and increased cytokine production, consistent with IFN-driven adaptive activation. In IL-33–responsive melanoma models, decitabine cooperated with IL-33 to elevate PD-1 expression and sensitize tumors to PD-1 blockade [186]. Both the previously described antigen upregulation as well as immune checkpoint expression alteration on CD4^+^ and CD8^+^ T cells have been observed simultaneously in head and neck squamous cell carcinoma in response to decitabine treatment [187]. Collectively, preclinical data support HMAs as agents that enhance tumor antigen presentation through IFN induction and influence T cell activation and cytokine production.

### 5.2. Clinical Applications of Viral Mimicry with Immunotherapy

Clinical trials have evaluated HMAs in combination with ICB therapy in AML and its precursor, myelodysplastic syndrome (MDS). Bone marrow disorders such as MDS have been prominent candidates for combination therapy because previous studies showed that MDS patients have an inherent upregulation of PD-L1, PD-L2, and CTLA-4 in their CD34^+^ bone marrow cells compared to chronic myelomonocytic leukemia (CMML) and AML patients, although all three display higher immune checkpoint expression compared to healthy bone marrow [188]. These studies also found that the immune checkpoint upregulation was further enhanced with HMA treatment, making immune checkpoint blockade a more potent target and suggesting HMA and ICB combination therapy to be a more viable option. In higher-risk MDS, clinical trials combining PD-1 inhibitors with HMA reported promising efficacy [189]. Further analysis of these clinical trial patients showed a negative correlation between T cell exhaustion markers, such as TIM-3 and PD-1, and overall response rate (ORR), indicating that this combination treatment mitigated T cell exhaustion, leading to better clinical outcomes. However, response rates were higher in treatment-naïve patients, whereas HMA or ICB therapy-refractory patients demonstrated reduced clinical efficacy [190]. One multicenter trial also observed limited efficacy with guadecitabine and atezolizumab in relapsed/refractory (r/r) MDS and CMML patients, noting non-severe immune-related adverse events [191]. This trial hypothesized that refractory patients, specifically those refractory to HMAs, have acquired HMA resistance due to unintentional T cell exhaustion caused by prior HMA treatment. Therefore, although combination therapy provides a promising option, it will be inherently less effective in HMA-refractory patients compared to HMA-naïve.

However, for AML patients, this combination of HMAs and ICB therapy has been tested extensively. Particularly in r/r patients, clinical trials report improvements in ORR and progression-free survival (PFS). Furthermore, fewer or milder immune-related adverse events were reported, indicating that the safety and tolerability of this combination are high [192,193]. A phase II study of azacitidine plus nivolumab identified increased CTLA-4 expression on the surface of patients’ CD4^+^ T effector cells as a treatment-associated biomarker, aligning with a previously mentioned preclinical study and indicating that this upregulation is preserved from in vitro to patient response [194]. These findings support the feasibility and tolerability of combining epigenetic therapy and checkpoint inhibitors in hematological diseases.

In classic Hodgkin’s lymphoma, decitabine combined with camrelizumab was evaluated in r/r disease. Combination therapy was associated with prolonged PFS and increased peripheral central memory T cells compared to camrelizumab alone [195]. ICB therapy-naïve patients were more likely to achieve complete response (CR) with this combination compared to camrelizumab alone [196]. The combination therapy could potentially reverse resistance to ICB therapy in refractory cancers. In a follow-up study for patients resistant to decitabine and camrelizumab treatment, the addition of HDAC inhibitor chidamide produced clinical responses, suggesting that layered epigenetic modulation may enhance checkpoint responsiveness [197].

Clinical translation has also extended to solid tumors, although with less observed efficacy. One example of this is a non-small cell lung cancer (NSCLC) phase I dose-escalation study, where guadecitabine was combined with pembrolizumab. Although ORR was modest, durable disease control was achieved in a fraction of heavily pretreated patients. There was also an observed reduction in L1 DNA methylation in blood and tumor samples, consistent with pharmacologic hypomethylation [198]. Increased intratumoral effector T cells were observed in a subset of responding patients, and individuals with clinical benefit exhibited higher baseline inflammatory signatures, supporting biologic activity of epigenetic priming in selected contexts. In metastatic melanoma refractory prior to immunotherapy, azacitidine plus carboplatin before avelumab rechallenge restored anti–PD-L1 activity in a subset of patients, accompanied by increased HLA class I transcription [199]. Similarly, in anti-PD-1 refractory head and neck squamous cell carcinoma, low-dose azacitidine combined with PD-1 and CTLA-4 blockade increased intratumoral IFN-γ and PD-L1 expression while improving survival outcomes on rechallenge [200]. A summary of current clinical combinations integrating viral mimicry inducers with immunotherapies across hematologic and solid malignancies is provided in Table 2.

Hematological malignancies and liquid tumors have been shown to be more responsive to combination therapy with HMAs and ICB compared to solid tumors. This disparity has been directly observed when comparing studies analyzing RE demethylation in solid and liquid tumor clinical trials testing HMAs. One Phase II study using an oral HMA called CC-468 in combination with durvalumab for colorectal, high-grade serous ovarian cancer and ER+ breast cancer [201] demonstrated that the treatment was tolerable, though paired tumor biopsies showed no significant change in L1 demethylation and no clinical responses were observed. Conversely, guadecitabine was tested in treatment-naïve AML patients, and a 17–25% drop in L1 methylation was observed [202]. This re-expression of L1 was accompanied by a composite CR rate of over 50%. Interestingly, patients on a ten-day dosing schedule experienced more robust and durable L1 demethylation compared to patients on a five-day schedule, indicating that dose cycling is one method of temporal control that can be implemented to maximize the effect of viral mimicry. Therefore, we can attribute the difference in response to HMAs between solid and liquid tumors to several reasons. One is that liquid tumors are more accessible, making the hypomethylation induced by HMAs more uniform. Secondly, the type I IFN response is actively repressed in AML, making viral mimicry more effective, as it can reverse this IFN dampening and elicit a direct cytotoxic effect, along with synergizing with ICB therapy [203].

In terms of safety, across clinical trials the combination of HMAs and ICB therapy is relatively well-tolerated, with adverse events comparable to those observed during monotherapy. The most frequent adverse events in solid and hematologic tumors included nausea, fatigue, and febrile neutropenia. Overall, these studies demonstrate that HMAs can function as immunologic priming agents in hematological and solid tumors by enhancing IFN-associated transcription and restoring antigen presentation to increase effector lymphocyte infiltration in tumors treated with checkpoint blockade, although their effectiveness is highly dependent on tumor accessibility and the level of inflammatory response.

### 5.3. Viral Mimicry Integration with Engineered Cell Therapies

**Figure 4:**
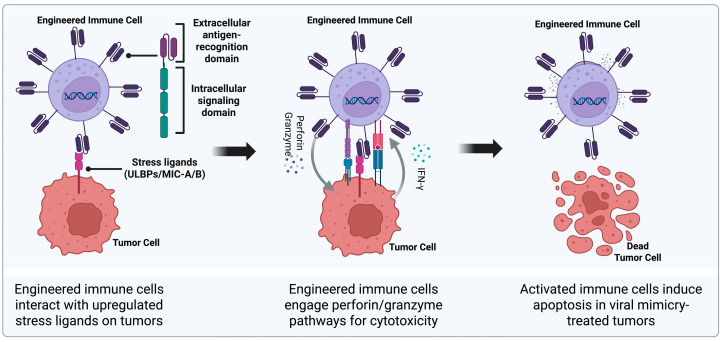
**CAR-engineered immune cell targeting of viral mimicry–induced stress ligands**. Synthetic receptors on CAR-engineered immune cells recognize stress ligands upregulated on viral mimicry–treated tumor cells, promoting tumor-specific cytotoxicity and apoptosis. Created in BioRender. Kirkland, A. (2026). https://BioRender.com/3ud0lsm (accessed on 27 March 2026).

Native lymphocyte responses frequently fail in solid and immune-excluded tumors due to limited trafficking, impaired antigen presentation and progressive dysfunction under sustained inflammatory signaling. Chimeric antigen receptor (CAR) platforms address several of these barriers by redirecting cytotoxic lymphocytes toward defined surface antigens to enable tumor recognition independent of classical MHC restriction (Figure 4). When these targets are preferentially expressed or induced on malignant cells, engineered recognition can improve tumor specificity and reduce off-target immune activity. This feature is particularly relevant in tumors reshaped by viral mimicry, where innate immune activation remodels a cold TME and creates an inflammatory selection pressure. In principle, this makes CAR therapy well suited for tumors in which viral mimicry increases immune visibility but endogenous immune priming remains incomplete.

The relationship between viral mimicry and engineered cell therapy is not uniformly beneficial. Viral mimicry may improve tumor recognition by CAR-T cells, CAR-NK cells, and endogenous immune populations. However, the same antiviral signaling state can also induce PD-L1, HLA class I, HLA-E, and other inhibitory programs that limit immune killing. Sustained IFN exposure may also impose selective pressure on tumors, as observed in melanoma where resistant clones persisted after inflammatory elimination of susceptible populations [204]. The strongest evidence includes studies that test HMAs, epigenetic priming, innate immune agonists, and engineered CAR platforms. These studies support the feasibility of integrating viral mimicry-adjacent biology with engineered cell therapy, but they also reveal key translational gaps. Many do not measure retroelement derepression or tumor-localized ISG responses. As a result, the current literature supports epigenetic remodeling as a primer for CAR therapy rather than a causal mechanism.

#### 5.3.1. Epigenetic Priming and CAR-T Cell Therapy

CAR-T cells are the most clinically mature engineered cell platform, with major success in hematologic malignancies and continued development in solid tumors [205,206,207,208,209]. Their central advantage is the ability to recognize surface antigens independently of endogenous TCR specificity and classical MHC antigen presentation [196]. This feature is relevant in tumors exposed to inflammatory selection pressure, where antigen presentation defects, reduced HLA expression, and clonal immune escape can limit native T cell recognition [195,196,197]. Second- and third-generation CAR designs improved early platforms by incorporating CD28 or 4-1BB co-stimulatory domains, which enhance proliferation, persistence, and effector function [199]. Newer designs address additional barriers through safety switches, such as HSV1-tk or iCaspase9, checkpoint disruption, cytokine armoring, multi-antigen recognition, and logic-gated control systems [198,210,211,212]. These features are clinically relevant in TMEs, where engineered cells must maintain cytotoxic functions despite inhibitory cues.

Epigenetic priming provides the clearest current bridge between viral mimicry biology and engineered T cell therapy. HMAs further interface with CAR platforms by reshaping both tumor antigenicity and T cell epigenetic states. In AML models, decitabine treatment upregulated the CTA NY-ESO-1 and improved the cytotoxicity and memory phenotype of NY-ESO-1–specific TCR-T cells, maintaining effector function over time [212]. Although this study used TCR-T rather than CAR-T cells, it demonstrates that epigenetic therapy can increase antigen-dependent susceptibility to adoptive T cell therapy. In CD123-directed CAR-T cells, decitabine reduced DNMT1 and DNMT3A expression and enriched naïve and early memory transcriptional signatures that enhanced anti-leukemic activity in xenograft models [213,214]. Low-dose decitabine similarly improved CAR-T persistence, cytokine production, recall activity, and remodeled exhaustion-associated loci in B cell leukemia and lymphoma models [215]. Early-phase clinical protocols in relapsed or refractory diffuse large B-cell lymphoma now incorporate low-dose decitabine before or during infusion of CD19- or tandem CD19/CD22-directed CAR-T cells to deepen responses and extend remission [216]. These studies support the concept that HMAs can act on both tumor cells and engineered T cells, but they do not uniformly prove that viral mimicry is the dominant mechanism.

Epigenetic priming may also help address antigen escape. In multiple myeloma, GPRC5D loss after CAR-T therapy can occur through promoter hypermethylation, suggesting that CAR pressure can select for antigen-low or antigen-silenced tumor states [217]. In DNMT3A-mutated AML, decitabine increased CD44v6 surface expression and improved CD44v6 CAR-T recognition, cytotoxicity, and persistence [218,219]. Similar priming effects have been observed in solid tumors. In ovarian carcinoma, decitabine increased CSPG4 expression, enabling effective targeting by CSPG4-specific CAR-T cells [220]. In bladder cancer, decitabine enhanced sensitivity to EGFR- and CD44v6-directed CAR-T cytotoxicity compared with unprimed epithelial cells, although the effect was not explained solely by increased target antigen expression [221]. This distinction is important, as epigenetic therapy may improve CAR activity through antigen induction, but it may also alter tumor survival pathways and cytokine signaling more broadly.

Clinical studies combining HMAs with CAR-T therapy support feasibility, but they need to be further investigated from a viral mimicry perspective. In r/r B-ALL, decitabine consolidation after CD19/CD22 CAR-T therapy improved overall and leukemia-free survival compared with standard maintenance strategies [222]. Decitabine has also been incorporated into lymphodepletion before CD19/CD22 bispecific CAR-T infusion, with favorable outcomes in r/r B-ALL [223]. A similar benefit was observed in diffuse large B-cell lymphoma, where decitabine-primed tandem CD19/CD22 CAR-T therapy produced encouraging efficacy signals [224]. Multimodal epigenetic priming has also been reported in high-risk settings. A case report in refractory primary CNS lymphoma described long-term complete remission after decitabine-primed tandem CD19/CD22 CAR-T therapy followed by PD-1 and BTK inhibitor maintenance [225]. These clinical studies show that epigenetic conditioning can be integrated into CAR-T treatment schedules, but they generally measure response, survival, and persistence rather than direct viral mimicry biomarkers.

This limitation should shape how the field interprets these data. HMAs can induce viral mimicry, but they also affect many nonviral mimicry pathways. Without measurements of RE activation, dsRNA accumulation, cytosolic nucleic acid sensing, type I IFN signaling, and tumor-localized immune remodeling, improved CAR-T outcomes cannot be attributed specifically to viral mimicry. Future trials should therefore pair clinical response data with pharmacodynamic markers that distinguish broad demethylation from viral mimicry states.

Viral mimicry-conditioned tumors may also require CAR-T products engineered to resist inhibitory feedback. IFN-rich TMEs can upregulate PD-L1 and other checkpoint ligands that suppress T cell function. CRISPR/Cas9-mediated PD-1 disruption enhanced mesothelin-targeted CAR-T proliferation, cytokine secretion, cytotoxicity against PD-L1-expressing targets, and tumor control in preclinical models [216]. Allogeneic CD19 CAR-T cells with CRISPR-disrupted PD-1 also showed improved functional fitness and persistence [216,226]. Cytokine-armored CAR-T cells provide another strategy. IL-18-secreting CAR-T cells showed enhanced expansion, antitumor activity, and activation of endogenous immune responses [210,211]. Together, these approaches reflect efforts to engineer CAR-T cells capable of maintaining activity within inflammatory and checkpoint-rich environments shaped by viral mimicry and other immunomodulatory therapies.

#### 5.3.2. Viral Mimicry and CAR-NK Therapies

CAR-NK cells provide a complementary strategy for tumors shaped by antiviral signaling. Unlike autologous CAR-T cells, CAR-NK cells can be generated from healthy donors, umbilical cord blood, induced pluripotent stem cells, or established cellular platforms, supporting off-the-shelf manufacturing and reduced production time [206,214,215,216]. CAR-NK platforms may also reduce the risk of graft vs host disease, severe cytokine release syndrome (CRS), and neurotoxicity compared with CAR-T therapy, although clinical experience remains more limited [205,214,227]. These features are relevant when considering combinations with viral mimicry-inducing agents, which may increase inflammatory toxicity if not properly controlled.

The biological rationale for CAR-NK therapy differs from CAR-T therapy. Viral mimicry can increase stress ligand expression and innate immune cues that align with NK cell recognition. NKG2D ligands, including MICA, MICB, and ULBP family members, are stress-regulated surface molecules that can mark malignant cells for NK cell recognition [228]. This ligand family provides a conceptual link between viral mimicry and CAR-NK therapy, but direct evidence that viral mimicry reliably induces these ligands across tumor contexts remains limited. CAR-NK constructs targeting MICA/B have shown enhanced cytotoxicity against MICA/B-expressing tumor cells, supporting stress ligand-directed CAR-NK therapy as a potential strategy when tumors display these ligands [229,230]. This approach could be relevant to viral mimicry-conditioned tumors if stress ligand expression is increased, but this connection should be tested directly in each tumor and treatment context.

IFN-conditioned tumors may also become less susceptible to NK cell-mediated killing through increased HLA class I and HLA-E expression. The HLA-E/NKG2A axis functions as an inhibitory checkpoint on NK cells and CD8^+^ T cells, and IFN-γ has been linked to increased HLA-E expression in tumor settings [231,232]. Inflammatory signaling may increase tumor visibility while also reinforcing inhibitory pathways that restrain NK cell cytotoxicity [233,234]. Engineering strategies that disrupt inhibitory recognition or convert HLA-E engagement into activating signals may therefore be required.

Preclinical studies further support integration of viral mimicry with NK cell-based therapies. HMAs and histone-modifying drugs increased NKG2D ligand expression and altered NK cell differentiation and function in hematologic malignancies in a dose-dependent manner [235]. Activation of the cGAS–STING pathway enhanced NK cell-mediated antitumor immunity, sustained TCF-1^+^ progenitor-like NK cell populations, and increased tumor infiltration [236,237,238]. In pancreatic cancer models, intratumoral delivery of cGAMP enhanced CAR-NK antitumor activity compared with CAR-NK therapy alone [239]. DNMT inhibition can also restore cGAS–STING signaling and activate RIG-I/MDA5–MAVS pathways in tumor cells, although this has not yet been established as a CAR-NK-specific mechanism [240]. Separately, DNMT1 inhibition improved cytokine-induced memory-like NK cell activity through increased autophagy, suggesting that epigenetic modulation can influence NK cell-intrinsic fitness [241].

Despite this rationale, CAR-NK integration with viral mimicry remains largely preclinical. Current studies support the feasibility of CAR-NK manufacturing from multiple sources, stress ligand-directed targeting, cytokine-supported persistence, and innate agonist-mediated enhancement of CAR-NK activity. However, they do not yet define the optimal NK cell source for IFN-conditioned tumors, establish whether viral mimicry improves CAR-NK persistence in vivo, or determine whether stress ligand-directed targeting can overcome tumor heterogeneity without unacceptable off-tumor effects. It also remains unclear whether viral mimicry should be induced before CAR-NK cell infusion, delivered locally during CAR-NK cell activity, or used as maintenance to preserve a targetable tumor state. These questions are central because durable NK cell-mediated tumor control requires sustained recognition and resistance to inhibitory feedback.

#### 5.3.3. Translational Gaps and Clinical Requirements

Although the available studies support the feasibility of combining epigenetic priming and engineered cell therapy, they do not yet define a mature clinical framework for viral mimicry–CAR integration. The central challenge is control. Viral mimicry must be induced strongly enough to increase tumor visibility, but not so broadly that it causes systemic inflammation or chronic IFN-driven immune dysfunction.

Delivery remains the first unresolved variable. Systemic HMAs are clinically accessible and have already been incorporated into CAR-T protocols, but their broad activity across malignant, immune, stromal and hematopoietic compartments complicates the mechanistic interpretation [212,213,215,222,223,224,225]. More direct viral mimicry strategies, such as STING agonists or synthetic nucleic acid mimetics, may provide stronger pathway control, but they will likely require local or tumor-targeted delivery to limit systemic toxicity [238,239,240]. Preclinical cGAMP delivery provides one proof-of-concept for this approach, but equivalent delivery strategies remain underdeveloped for most CAR-based combinations [239].

In addition to delivery, the timing of drug administration remains another gap in knowledge. Viral mimicry could be induced before infusion to increase tumor targetability, during lymphodepletion to remodel the immune environment, after infusion to sustain antigen expression, or intermittently to limit antigen escape. Each schedule has distinct risks. Early induction may also upregulate PD-L1 or HLA-E prior to immune activation. Maintenance dosing may preserve antigen expression but promote chronic IFN signaling and effector dysfunction. Current clinical decitabine-CAR-T studies demonstrate feasibility across several schedules, but they do not establish the optimal sequence for viral mimicry induction [214,215,216,217].

Finally, biomarker-defined trials will be essential to distinguish therapeutic tumor conditioning from nonspecific immune activation. If viral mimicry increases a CAR target or stress ligand on normal tissues, it could worsen on-target, off-tumor toxicity. Future studies should therefore compare paired tumor and normal tissue responses using pharmacodynamic markers of viral mimicry, target induction, and inflammatory toxicity.

Together, these gaps suggest that viral mimicry should be developed as a controlled tumor-conditioning strategy rather than a generalized immune stimulant. Its clinical value will depend on localized delivery or tumor cell-specific activation, biomarker-guided timing, and CAR designs that account for both the activating and suppressive consequences of antiviral signaling.

## 6. Conclusions and Future Perspectives

Viral mimicry has reframed REs from passive genomic remnants into active participants in tumor immune regulation. The epigenetic programs that preserve malignant identity can also conceal nucleic acid species capable of triggering antiviral immunity. The central question moving forward is whether this hidden immunogenicity can be controlled with enough precision to produce durable tumor clearance.

This question is especially important because viral mimicry operates through pathways that can support both immune recognition and immune adaptation. IFN signaling can increase tumor visibility, but persistent activation can also reinforce checkpoint expression, immune dysfunction, and clonal escape. The therapeutic challenge is therefore not simply to induce viral mimicry, but to define the conditions under which antiviral signaling becomes productive rather than suppressive.

Future progress will depend on strategies that control the timing, administration, and dosage of viral mimicry induction. This will require biomarkers that identify tumors with intact antiviral sensing, delivery approaches that limit systemic inflammation, and combinations that pair tumor priming with immune effectors capable of acting on the induced state. Engineered immune platforms, including CAR-T and CAR-NK therapies, may broaden the therapeutic use of viral mimicry-primed tumors by innate immune activation. Modalities that rely on antigen spreading, such as neoantigen vaccines, may further extend this strategy by converting transient antiviral signaling into durable adaptive immune recognition. For patients with limited options, the promise of viral mimicry lies in making resistant tumors visible to immune recognition when current therapies fail.

## Figures and Tables

**Table 1:** Summary of viral mimicry inducers, pathways, biomarkers, and clinical status.

Inducer Class	Representative Agents	Mechanism of Viral Mimicry Induction	Dominant Sensing Pathway(s)	Biomarkers	Clinical Status	Key References
DNMT inhibitors	Azacitidine, Decitabine	Demethylation of endogenous REs leads to dsRNA accumulation	RIG-I, MDA5 → MAVS → TBK1/IKK → IRF3/7	ERV transcripts, dsRNA signal, IFN-I/III signature, CXCL9/10, MHC-I, TAP1/2	FDA-approved for hematologic malignancies; multiple immunotherapy combinations under clinical investigation	Roulois 2015 [24]; Chiappinelli 2015 [27]; Peng 2015 [103]; Ganesan 2019 [109]; De Cubas 2020 [110]
Next-generation DNMT-targeting agents	Guadecitabine, DNMT1-selective inhibitors	Sustained repeat derepression through enhanced DNMT inhibition	RIG-I/MDA5-driven IFN signaling	ERV RNA, CpG hypomethylation at repeat loci, ISG induction	Guadecitabine in oncology trials; DNMT1-selective inhibitors remain largely preclinical	Griffiths 2013 [104]; Pappalardi 2021 [105]; Mehdipour 2021 [106]; Jang 2023 [107]; Amaro 2023 [108]
H3K9 methyltransferase axis disruption	SUV39H1 pathway targeting	Loss of H3K9me3-mediated repeat silencing results in reactivation	dsRNA- and dsDNA-sensing pathways	Reduced H3K9me3 at repeats, LINE-1 and ERV RNA, ISG expression	Preclinical oncology studies	Bulut-Karslioglu 2014 [60]; Lu 2019 [112]; Shen 2021 [113]; Niborski 2022 [114]
SETDB1 loss or inhibition	SETDB1 targeting strategies	Impaired H3K9me3 deposition at ERVs promotes IFN activation	RIG-I/MDA5 pathways	ERV expression, IFN response genes, enhanced antigen processing genes	Preclinical tumor immunity models	McGeary 2025 [115]
EZH2 inhibition (combination context)	EZH2 inhibitors	Relief of Polycomb-mediated repression that amplifies repeat transcription in DNMTi-primed tumors	IFN signaling amplification	H3K27me3 reduction, augmented ISG and chemokine expression	Clinically approved class: viral mimicry explored in combination strategies	Chomiak 2024 [117]
HDAC inhibition (combination context)	Pan-HDAC inhibitors	Chromatin accessibility increases TE transcription, particularly with DNMT inhibition	dsRNA-dependent IFN activation	ERV-derived transcripts, ISGs, antigen presentation genes	FDA-approved in select cancers; immunotherapy combinations ongoing	Goyal 2023 [116]
Synthetic dsRNA agonists	Poly(I:C), poly-ICLC	Exogenous dsRNA mimics directly activate RNA sensors	TLR3, MDA5 → IRF3/NF-κB	Acute IFN-I induction, CXCL10, dendritic cell activation markers	Early-phase clinical studies, intratumoral and vaccine-adjuvant settings	Sultan 2020 [122]; De Waele 2021 [123]
RIG-I agonists	5′-triphosphate RNA, SLR14	Direct activation of RIG-I independent of chromatin remodeling	RIG-I → MAVS → TBK1 → IRF3	Type I IFN peak kinetics, ISGs, enhanced antigen presentation	Preclinical and early translational development	Jiang 2019 [124]; Jiang 2023 [125]; Jacobson 2019 [126]; Wu 2017 [127]
STING agonists	ADU-S100 and related compounds	Direct STING activation, bypassing endogenous DNA generation	STING → TBK1 → IRF3 and NF-κB	IFN-I signature, CXCL9/10, immune infiltration markers	Preclinical and early clinical development; combination-focused	Lee 2021 [128]; Falahat 2023 [129]

**Table 2:** Clinical combinations of viral mimicry and immunotherapies.

Cancer Type	*n*	Phase	Hypomethylating Agent	Immunotherapy	Clinical Outcomes	Trial Code	Citation
Various solid tumors (NSCLC, cervical, cholangiocarcinoma, colorectal, breast, prostate, ovarian, mesothelioma, renal)	30	I	Guadecitabine	Pembrolizumab	ORR of 7%, and 37% achieved PFS	NCT02998567	Papadatos-Pastos, 2022 [198]
Metastatic melanoma	20	II	Azacitidine	Avelumab	~90% of patients who underwent 2 cycles of priming achieved disease stabilization or partial response	ACTRN12618000053224	Van der Westhuizen, 2022 [199]
Metastatic head and neck squamous cell carcinoma	12	I	5-azacytidine	Durvalumab, Tremelimumab	58% of patients had prolonged overall survival of >12 months	NCT03019003	Qin, 2025 [200]
Metastatic urothelial carcinoma	21	II	Guadecitabine	Atezolizumab	OS of 8.6 months, median PFS of 3 months	NCT03179943	Jang, 2023 [107]
Myelodysplastic syndrome	53	II	Decitabine	Sintilimab	ORR of 77%, CR of 26%	ChiCTR210044393	Wang, 2024 [189]
Myelodysplastic syndrome	37	II	Azacitidine	Pembrolizumab	HMA-naïve patients: ORR 76%; HMA-failure: ORR 25%	NCT03094637	Chien, 2021 [190]
r/r myelodysplastic syndrome, chronic myelomonocytic leukemia	33	I/II	Guadecitabine	Atezolizumab	ORR of 33%, mOS 15.1 months	NCT02935361	O’Connell, 2022 [191]
r/r AML	37	II	Decitabine, azacytidine	Tislelizumab	30% no response, 58% CR/CRi, 11% achieved PR	NCT04541277	Zhou, 2025 [140]
r/r AML	27	II	Decitabine, azacitidine	Tislelizumab	ORR of 63%	NCT04541277	Gao, 2023 [193]
r/r AML	70	II	Azacitidine	Nivolumab	ORR of 33%; higher ORR in HMA-naïve patients than in HMA pre-treated	NCT02397720	Daver, 2019 [194]
r/r Hodgkin’s lymphoma	42	II	Decitabine	Camrelizumab	CR of 79% in HMA + ICB vs. 32% in ICB only	NCT02961101	Liu, 2021 [195]
r/r Hodgkin’s lymphoma	86	II	Decitabine	Camrelizumab	In ICB-naïve patients, CR was 32% vs. combination therapy 71% CR	NCT03250962	Nie, 2019 [196]
r/r Hodgkin’s lymphoma	52	II	Chidamide, decitabine	camrelizumab	ORR of 94%; CR of 50%; all patients previously displayed ICB resistance, which was mitigated somewhat by triplet therapy	NCT04233294	Nie, 2024 [197]

## Data Availability

No new data were created or analyzed in this study. Data sharing is not applicable to this article.

## Abbreviations

The following abbreviations are used in this manuscript:

APC	Antigen Presenting Cell
CAFs	Cancer Associated Fibroblasts
CAR	Chimeric Antigen Receptor
CAR-NK	Chimeric Antigen Receptor Natural Killer Cells
CXCL	C-X-C Motif Ligand
cGAS	Cyclic GMP-AMP Synthase
CTLA-4	Cytotoxic T-Lymphocyte Antigen 4
DNMT	DNA Methyltransferase
DNMTi	DNA Methyltransferase Inhibitor
dsDNA	Double-Stranded DNA
dsRNA	Double-Stranded RNA
ECM	Extracellular Matrix
ERVs	Endogenous Retroviruses
EZH2	Enhancer of Zeste Homolog 2
HDAC	Histone Deacetylase
HLA	Human Leukocyte Antigen
ICB	Immune Checkpoint Blockade
IFN	Interferon
IFN-γ	Interferon Gamma
IKK	IκB kinase
IRF3	Interferon Regulatory Factor 3
IRF7	Interferon Regulatory Factor 7
ISGs	Interferon-Stimulated Genes
JAK-STAT	Janus Kinase–Signal Transducer and Activator of Transcription
LAG-3	Lymphocyte Activation Gene 3
LINE-1	Long Interspersed Nuclear Element-1
LSD1	Lysine-Specific Demethylase 1
MAVS	Mitochondrial Antiviral-Signaling Protein
MDA5	Melanoma Differentiation-Associated Protein 5
MDSCs	Myeloid-derived Suppressor Cells
MHC-I	Major Histocompatibility Complex Class I
MICA/B	MHC Class I Polypeptide-Related Sequence A/B
NF-κB	Nuclear Factor Kappa B
NK	Natural Killer Cells
NKG2D	Natural Killer Group 2D Receptor
PD-1	Programmed Cell Death Protein 1
PD-L1	Programmed Cell Death Ligand 1
PRRs	Pattern Recognition Receptors
REs	Repetitive Elements
RIG-I	Retinoic Acid-Inducible Gene I
r/r	Relapse/refractory
SINEs	Short Interspersed Nuclear Elements
STING	Stimulator of Interferon Genes
SUV39H1	Suppressor of Variegation 3–9 Homolog 1
TBK1	TANK-Binding Kinase 1
TEs	Transposable Elements
TME	Tumor Microenvironment
Tregs	Regulatory T cells
ULBPs	UL16 Binding Proteins
TILs	Tumor-Infiltrating Lymphocytes
TLR3	Toll-Like Receptor 3
TYK2	Tyrosine Kinase 2
